# “To me, it was overwhelming”: a mixed methods study of maternal healthcare in a marginalised community in New York City, guided by the person-centered care framework for reproductive health equity

**DOI:** 10.1080/26410397.2026.2632452

**Published:** 2026-02-20

**Authors:** Khadija R. Jones, Nandini Choudhury, Mary Archana Fernandez, George Hagopian, Rachel Schwartz, Adiba Chowdhury, Sushmita Diyali, Keeley McNamara, Hugo Teo, Payal Ram, Andrea Archer, Amanda Misiti, Teresa Janevic, Sheela Maru

**Affiliations:** a Program Manager, Arnhold Institute for Global Health, Icahn School of Medicine at Mount Sinai, New York, NY, USA. *Correspondence*: khadija.jones21@gmail.com; b Data Analyst, Arnhold Institute for Global Health, Icahn School of Medicine at Mount Sinai, New York, NY, USA; c Director Family Health Services, South Asian Council for Social Services, New York, NY, USA; d Chief of Gynecologic Oncology, New York City Health + Hospitals, New York, NY, USA; e Senior Director Maternal Child Health, Public Health Solutions, New York, NY, USA; f Senior Manager Family Support Services, South Asian Council for Social Services, New York, NY, USA; g Senior Manager Health Services, South Asian Council for Social Services, New York, NY, USA; h Certified Nurse Midwife, New York City Health + Hospitals, New York, NY, USA; i Senior Consultant, New York City Health + Hospitals, New York, NY, USA; j Research Program Coordinator, Arnhold Institute for Global Health, Icahn School of Medicine at Mount Sinai, New York, NY, USA; Program Coordinator, New York City Health + Hospitals, New York, NY, USA; k UX Designer, Arnhold Institute for Global Health, Icahn School of Medicine at Mount Sinai, New York, NY, USA; l Associate Director of Communications and Global Partnerships, Arnhold Institute for Global Health, Icahn School of Medicine at Mount Sinai, New York, NY, USA; m Epidemiologist, Arnhold Institute for Global Health, Icahn School of Medicine at Mount Sinai, New York, NY, USA; n Director of the NYC partnership, Arnhold Institute for Global Health, Icahn School of Medicine at Mount Sinai, New York, NY, USA. [Obstetrician Gynaecologist and Director of Global Health] New York City Health + Hospitals, New York, NY, USA; Associate Professor, Department of Obstetrics, Gynaecology and Reproductive Science, Icahn School of Medicine at Mount Sinai, New York, NY, USA

**Keywords:** person-centered care, maternal healthcare, COVID-19, health disparities, health inequalities, mixed-methods, New York City, reproductive health equity, social determinants of health, healthcare utilisation

## Abstract

The Person-Centered Care Framework for Reproductive Health Equity (PCC) elucidates drivers of health disparities: community determinants, health-seeking behaviours and quality of care. Limited studies assess person-centered maternal healthcare in underserved populations. Racial and ethnic disparities in maternal health were exacerbated by the COVID-19 pandemic. We applied PCC to evaluate factors influencing maternal healthcare at two public hospitals in NYC before and during the pandemic. We conducted mixed-method, community-engaged research using PCC. A cross-sectional study using EHR data from 5330 pregnant individuals in 2019 assessed factors related to inadequate maternity care utilisation. Qualitative research in 2020 explored perceptions of maternal health equity, barriers, and healthcare quality through 17 in-depth interviews and five focus group discussions with postpartum women, clinicians, and community-organisation staff. Among 3181 women, 90% had public insurance, and 95% were people of colour. Using the Adequacy of Prenatal Care Utilisation index, 1648 (51.8%) received no or inadequate prenatal care and 1267 (40%) lacked postpartum care. Women aged 18–24, Black women, Arabic-speaking women and those who used tobacco during pregnancy appeared more likely to experience inadequate care. Qualitative data identified community-level determinants, namely health literacy and economic status. Health-seeking barriers included social distancing, telehealth and immigration status. Quality of care issues included disruptions in healthcare delivery, patient-provider experience and continuity of care. Findings indicate disparities in maternal healthcare utilisation, which are likely downstream effects of broader social inequities. Addressing these disparities requires rights-based, community-informed policies that guarantee equitable, respectful and accessible maternal care for all.

## Plain language summary

The Person-Centered Care Framework for Reproductive Health Equity (PCC) aims to improve maternal care by focusing on the needs and experiences of people giving birth. It looks at issues like access to and use of healthcare, community support and the quality of care received. There has been limited use of this framework to assess reproductive health equity amongst marginalised groups outside of low- and middle-income countries. Since the COVID-19 pandemic made racial and ethnic disparities in maternal health worse, it is important to study its effects closely. We studied maternal healthcare at two NYC public hospitals. We used the PCC framework before and during the pandemic. In 2019, we collected data from 5330 pregnant people. We also interviewed new mothers, healthcare providers and community staff in 2020. Our research found that most of the 3181 new mothers were women of colour with public insurance. Many lacked postpartum care, and over half received poor prenatal care. Inadequate care seemed more common among younger women, Black women, Arabic-speaking women and women who smoked during pregnancy. Barriers to care included**:** (1) lack of health awareness, (2) financial hardships, (3) social isolation, (4) challenges with telehealth and (5) immigrant status. Problems with the quality of care included: (6) poor communication between patients and doctors and (7) not getting consistent care. The results show differences in how women use maternal healthcare, which are likely caused by larger social issues. To fix these disparities, we need policies that are equitable and involve the community.

## Résumé

Le cadre de soins centrés sur la personne pour l’équité en matière de santé reproductive permet d’expliciter les facteurs à l’origine des inégalités de santé: déterminants communautaires, comportements de recours aux soins et qualité des soins. Peu d’études évaluent les soins maternels centrés sur la personne au sein des populations sous-desservies. Les inégalités raciales et ethniques en matière de santé maternelle ont été exacerbées par la pandémie de COVID-19. Nous avons utilisé le cadre de soins centrés sur la personne pour évaluer les facteurs qui ont influencé les soins maternels dans deux hôpitaux publics dans la ville de New York avant et pendant la pandémie. À l’aide de ce cadre, nous avons mené une recherche à méthodes mixtes qui associait les communautés. Une étude transversale, se fondant sur les données des dossiers de santé électroniques de 5330 personnes enceintes en 2019, a évalué les facteurs relatifs à une utilisation inadéquate des soins de maternité. En 2020, une recherche qualitative a exploré les perceptions de l’équité en matière de santé maternelle, les obstacles et la qualité des soins au cours de 17 entretiens approfondis et de cinq discussions par groupe d’intérêt avec des femmes en postpartum, des cliniciens et des membres d’organisations communautaires. Parmi les 3181 femmes, 90% bénéficiaient d’une assurance maladie publique et 95% étaient issues de minorités ethniques. Selon l’indice d’adéquation du recours aux soins prénatals, 1648 (51,8%) avaient reçu des soins prénatals inadéquats ou pas de soins du tout; 1267 (40%) ne bénéficiaient pas de soins du postpartum. Les femmes âgées de 18 à 24 ans, les femmes noires, les femmes arabophones et celles qui avaient fumé pendant la grossesse semblaient plus à risque de recevoir des soins inadéquats. Les données qualitatives ont permis d’identifier des déterminants au niveau communautaire, à savoir les connaissances de base en santé et la situation économique. Parmi les obstacles à l’accès aux soins figuraient la distanciation sociale, la télémédecine et le statut migratoire. Les problèmes de qualité des soins incluaient les perturbations dans la prestation des soins, l’expérience patient-soignant et la continuité des soins. Les résultats indiquent des disparités dans l’utilisation des soins de santé maternelle, qui sont probablement les conséquences d’inégalités sociales plus générales. Pour corriger ces disparités, il est nécessaire de disposer de politiques fondées sur les droits et élaborées sous la direction des communautés, garantissant des soins maternels équitables, respectueux et accessibles à toutes les personnes.

## Resumen

El Marco de Atención Centrada en la Persona (ACP) para la Equidad en Salud Reproductiva aclara los factores que impulsan las disparidades en salud: los determinantes comunitarios, los comportamientos de búsqueda de servicios de salud y la calidad de la atención. Estudios limitados evalúan los servicios de atención materna centrada en la persona en poblaciones desatendidas. Las disparidades raciales y étnicas en salud materna fueron exacerbadas por la pandemia de COVID-19. Aplicamos la ACP para evaluar los factores que influyen en los servicios de salud materna en dos hospitales públicos de la Ciudad de Nueva York prepandemia y durante la pandemia. Realizamos una investigación de métodos mixtos con la participación comunitaria, utilizando ACP. Un estudio transversal que utilizó datos de Registros de Salud Electrónicos (RSE) de 5330 personas embarazadas en 2019 evaluó los factores relacionados con el uso de servicios de atención materna inadecuada. Una investigación cualitativa en 2020 exploró las percepciones de la equidad en salud materna, las barreras y la calidad de los servicios de salud por medio de 17 entrevistas a profundidad y cinco debates en grupos focales con mujeres posparto, profesionales médicos y personal de movilización comunitaria. Entre 3181 mujeres, el 90% tenía seguro médico público y el 95% era personas de colour. Utilizando el índice de la Adecuación de la Utilización de la Atención Prenatal, 1648 (51.8%) recibieron ninguna o inadecuada atención prenatal; 1267 (40%) carecían de atención posparto. Las mujeres de 18 a 24 años, las mujeres negras, las mujeres que hablan árabe y aquéllas que usaron tabaco durante el embarazo parecieron ser más propensas a recibir atención inadecuada. Los datos cualitativos identificaron los determinantes a nivel comunitario, principalmente la alfabetización en salud y el nivel socioeconómico. Entre las barreras a la búsqueda de servicios de salud figuraban el distanciamiento social, la telesalud y la situación migratoria. Algunos de los problemas con la calidad de la atención eran: las interrupciones en la prestación de servicios de salud, la experiencia paciente-prestador de servicios y la continuidad de la atención. Los hallazgos indican las disparidades en el uso de atención materna que probablemente son efectos posteriores de inequidades sociales más amplias. Para abordar estas disparidades se necesitan políticas basadas en los derechos e informadas por la comunidad, que garanticen atención materna equitativa, respetuosa y accesible a todas las personas.

## Background

In recent years, there has been a growing effort amongst community-based organisations, public health practitioners and government organisations to strengthen and improve maternal and reproductive healthcare as a means to end preventable deaths and advance health equity. Reproductive health is a basic human right, rooted in the universal principles of life, dignity, bodily autonomy, and the equitable opportunity to achieve optimal health and well-being.^1–3^ These rights are not conditional; they must be guaranteed to all people, regardless of race, ethnicity, socioeconomic status or immigration background. Yet, despite longstanding efforts to advance equity, significant disparities remain in reproductive healthcare access and outcomes, particularly for Black, Hispanic or Latinx, Asian, American Indian and Alaska Native, and Native Hawaiian or other Pacific Islander communities in the United States (US).^4,5^ From a rights-based perspective, these inequities reflect systemic violations that deny entire communities the opportunity to thrive. The COVID-19 pandemic further exacerbated these disparities, highlighting the urgent need for targeted interventions and policies to address systemic inequalities in healthcare access and delivery. A substantial body of research demonstrates a long history of racial and ethnic inequalities and disparities across several areas of reproductive health, including contraceptive use, sexually transmitted infection care, reproductive cancers, preterm deliveries, low-birth-weight neonates, and maternal morbidity and mortality.^4^ In 2019, the maternal mortality ratio among Hispanic women was 12.6 per 100,000 live births but rose notably during the pandemic reaching 18.2 per 100,000 live births in 2020 and 27.5 per 100,000 live births in 2021.^6^ A similar but more pronounced trend was observed among non-Hispanic Black women, whose maternal mortality rate increased from 44.0 in 2019 to 55.3 in 2020 and sharply rose to 69.9 in 2021 – 2.6 times higher than the rate among non-Hispanic White women (26.6).^7^ These alarming trends reflect the disproportionate impact of the pandemic on communities of colour and underscore how longstanding structural inequities continue to shape maternal health outcomes. Importantly, recent data indicate that more than 8 out of 10 (84%) of these pregnancy-related deaths are preventable, emphasising the urgent need to explore and address the underlying causes of these disparities through systemic and sustainable public health efforts.^5^

The roots of these disparities can be traced to a multifaceted interplay of factors, including variations in healthcare quality, underlying chronic conditions, structural racism, intergenerational trauma and implicit bias.^8^ Beyond individual-level risks, a growing body of research underscores the broader social and structural forces that create and sustain disproportionate risks for women of colour**.**^9^ These deeply entrenched inequities – including fragmented health insurance systems, discriminatory clinical practices and policies that neglect marginalised communities – reflect not just disparities in care but also systemic violations of reproductive rights and justice. For Black, Hispanic or Latinx, Asian, American Indian and Alaska Native, and Native Hawaiian or other Pacific Islander women, these factors intersect in ways that compound vulnerability across generations.^4^ The lived experiences of these communities are shaped not only by medical encounters but also by the cumulative impacts of housing instability, environmental exposures, economic insecurity and histories of disinvestment in their communities.^4^ Improving maternal health outcomes thus requires a shift from solely addressing clinical risk factors to tackling the broader social, economic and political determinants of health. This includes advocating for culturally responsive care models, investing in community-based solutions and implementing policies that explicitly address the legacy of racism and inequality embedded in the healthcare system. In doing so, we can move towards a more equitable maternal health landscape that upholds the rights, dignity, safety and well-being of all birthing people* and ensures that structural and systemic inequities are treated not only as health disparities but also as violations of human rights.

Notably, within the dynamic demographic landscape of New York City (NYC), where, in 2018, half of all births were to women born outside the US – it becomes imperative to examine maternal health care utilisation among immigrant populations.^10^ NYC’s large and diverse immigrant population, primarily from Latin America, Asia and the Caribbean, speaks over 200 languages and makes up approximately 36% of the city's population. This diversity presents both a rich cultural context and a complex set of challenges that demand tailored, culturally responsive strategies to address maternal health disparities and promote equitable outcomes.^10^ In NYC, healthcare delivery has long been plagued by racial inequities in maternal mortality, with Black women being nine times more likely to die of a pregnancy-related cause than White women.^11^ Decades of systemic racism – entrenched in and expressed through social determinants such as high rates of poverty, poor housing, unemployment and limited access to healthy and affordable food – have led patients to avoid or delay seeking care, be misdiagnosed or receive inappropriate treatment.^12^ In March 2020, New York City became the epicentre of the pandemic, which devastated the hospital and public health system, leaving immense economic and social damage. In addition to increased hospitalisations and deaths among pregnant women, maternal and child healthcare delivery was severely impacted as staffing was short, in-person prenatal and postpartum care visits were limited, and resources were scarce.^13^ Data from the CDC reveal that from March 2020 to January 2023, COVID-19 hospitalisations were 2.1 times higher for Black individuals compared to White Americans and 1.8 times higher for Hispanic individuals.^14^ These stark disparities reflect not only structural inequities but also systemic violations of human rights. The right to safe, respectful and accessible maternal healthcare is fundamental, and when marginalised communities are consistently denied this care, it represents a failure of the systems meant to protect and uphold their health, dignity and autonomy. These compounded impacts of racial injustice and pandemic-related disruptions underscore the urgency of addressing both immediate healthcare access and the broader systemic barriers that undermine maternal health, especially for immigrant and marginalised communities, whose rights to health and wellbeing must be safeguarded.

The Person-Centered Care Framework for Reproductive Health Equity (PCC) elucidates mechanisms for these health disparities and sheds light on avenues for fostering a more equitable and inclusive healthcare system.^2^ Originally developed to improve the quality of reproductive health care in low- and middle-income settings, the PCC framework centres the experiences and preferences of patients in clinical decision-making.^2,15^ Adapted from the definition by the Institute of Medicine, Sudhinaraset et al. define person-centred reproductive health care as: “Providing reproductive health care that is respectful of and responsive to individual women and their families’ preferences, needs and values, and ensuring that their values guide all clinical decisions”.^2^ Within the framework, three interrelated conceptual domains converge to advance reproductive health equity through person-centred care: (1) societal and community determinants of health equity, (2) women’s health-seeking behaviours and (3) facility-level factors.^2,15^ By centring the needs of patients, the PCC framework facilitates more nuanced analyses of the healthcare system and supports improvements in both care delivery and policy development. Our study builds on this framework to address the limited application of PCC in evaluating maternal care among underserved populations within high-income countries, specifically, individuals who are medically underserved (e.g. with limited access to quality healthcare), economically disadvantaged or socially marginalised. Situated within NYC’s public hospital system, which predominantly serves low-income, immigrant communities, our research underscores the importance of embedding person-centred principles into healthcare delivery as a means of advancing reproductive health equity and improving maternal health outcomes. We conducted a community-engaged research study using a triangulation design, convergent parallel approach, incorporating the components of the Person-Centered Care Framework. This study aimed to assess maternal healthcare delivery and identify potential barriers and gaps in care within a public healthcare delivery system in NYC. Our study spans both pre- and post-pandemic periods, enabling us to examine changes in healthcare delivery and rights-based experiences among marginalised populations in the context of the COVID-19 pandemic.

## Methods

### Study site

The public healthcare delivery system in New York City is intricate and robust, encompassing the NYC Department of Health and Mental Hygiene, the Health and Hospitals Corporation, academic centres and private healthcare providers, alongside numerous community-based organisations (CBOs). Our study site comprised two hospitals within NYC Health + Hospitals. NYC Health + Hospitals is the largest public healthcare system in the US, serving more than 1 million people annually, encompassing a diverse Latinx, Black non-Hispanic, Asian and immigrant population who face high rates of poverty and financial strain.^16,17^ We engaged closely with community-based organisations dedicated to serving the diverse sub-populations associated with the hospitals to conduct this study. The engaged CBOs included the South Asian Council for Social Services and Public Health Solutions. Our selection of these CBOs was informed by interactions with clinical providers engaged in primary care delivery, direct conversations with the CBOs, and a comprehensive internet search of local organisations providing services to the patient populations served by the hospitals. Notably, the participating CBOs play a pivotal role as primary providers of social services for the key immigrant groups within our catchment area.

### Study design

This study employed a triangulation design using a convergent parallel mixed-methods approach, wherein qualitative and quantitative data were collected during the same overall study period and analysed separately to provide a comprehensive understanding of the research question. Although both components were conducted in parallel, the quantitative data were retrospective, drawing on electronic health record data from a prior time period, while the qualitative data captured contemporaneous experiences related to changes during the COVID-19 pandemic. This design allowed for integration of retrospective and current perspectives to better understand the impact of COVID-19 on maternal healthcare.

#### Quantitative component

Quantitative data were obtained retrospectively from the hospitals’ electronic health records (EHR; EPIC System) spanning 2019 (pre-pandemic). The data focused on pregnant adults to examine healthcare utilisation and outcomes.

#### Qualitative component

Qualitative data were collected through key informant interviews and focus group discussions with hospital staff, CBO members and postpartum women residing in neighbourhoods served by the hospital. These discussions examined barriers and facilitators to equitable, comprehensive, and person-centred care across the maternal healthcare continuum within a predominantly immigrant population in NYC. The interviews and focus groups also explored societal and community determinants of maternal health equity and perceptions of care quality. To minimise bias and ensure diverse community perspectives, recruitment of postpartum women was conducted with support from our collaborating CBOs. Participants received a US$25 Amazon gift card as compensation for their time.

### IRB approval

This study was approved by the Institutional Review Board (IRB) of the Icahn School of Medicine at Mount Sinai (Study-20-0031) on 20 June 2020. Ethical approval covered the period from 20 June 2020 to 13 June 2024 and included permission to conduct a retrospective chart review of patient data from 1 January to 31 December 2019. Institutional approval from NYC Health + Hospitals was obtained on 3 May 2021 (STUDY00002675).

### Quantitative data collection

A query was used to retrospectively extract EHR data on all pregnancies among adults (≥18 years) registered at the two hospitals between January 1 and December 31, 2019. The extracted dataset included demographic data such as age, race, ethnicity, language, ZIP code and data on healthcare utilisation of recommended visits and screening.^18–20^ It also included key maternal and infant health outcomes of interest: hypertension, diabetes, obesity, substance use, and depression in pregnancy; live or still birth; birth weight; preterm birth; anaesthesia during birth; blood transfusion and 5-minute APGAR score.

### Qualitative data collection

Interview and focus group questions were developed guided by the PCC. We used a triangulated approach to qualitative data collection that included key informant interviews (KIIs) and focus group discussions (FDGs) to capture both institutional and community perspectives on maternal and child healthcare delivery. All qualitative data collection took place between August and September 2020. FGDs were conducted solely with postpartum women to foster peer dialogue and surface shared experiences of care. In contrast, KIIs – conducted with clinical staff and CBO representatives – provided scheduling flexibility and exploration of individual experiences, professional perspectives and system-level factors.

Postpartum women were recruited in partnership with our collaborating CBOs that had established relationships with families receiving services. Staff identified eligible individuals – women at least 18 years old who had delivered a live birth after 1 January 2020 and resided within the hospital’s catchment area. CBO partners referred postpartum clients to the study and supported the informed consent process using IRB-approved translated study materials and language-concordant staff to obtain verbal and written consent. Focus groups were organised by language spoken to ensure accessibility and participant comfort; five FGDs were conducted virtually in English, Spanish, Mandarin, Bengali and Nepali and were facilitated by CBO staff fluent in the corresponding languages.

Recruitment of clinical and CBO staff was conducted through email listservs and professional networks, including peer referrals and word-of-mouth outreach. Many CBO participants were referred collegially from within their own organisations. Outreach included information about the study’s goals, expectations and incentive details. While these methods relied on existing networks, efforts were made to ensure diversity across participant roles and experiences. 17 key informant interviews were conducted: nine with clinical staff (including an OB/GYN resident, physician assistant, attending OB/GYN, nurse administrator, midwife, primary care physician, clinical psychologist, licensed clinical social worker and director of nursing) and eight with representatives from our collaborating CBOs, including programme managers, translators, directors, community health workers, case managers and maternal health advocates.

All interviews and FGDs were conducted virtually via a HIPAA-compliant Zoom platform during the COVID-19 pandemic and lasted approximately 60 minutes. Participants joined from private spaces of their choosing. All postpartum participants had access to personal smartphones or internet-enabled devices and did not require a shared location to participate. KIIs were facilitated by a trained programme manager from the research team. FGDs conducted in Spanish, Mandarin, Bengali and Nepali were led by partnering CBO staff who were fluent in those languages. These facilitators signed letters of attestation confirming both their use of the approved interview guides and the accuracy of translated study materials. To ensure participant confidentiality, all sessions were held using secure links, and no personally identifying information was shared during discussions or recorded in transcripts. Additionally, participants were encouraged to use pseudonyms to further protect their identities.

### Quantitative data analysis

Descriptive statistics were used to summarise key characteristics of women who were identified as pregnant at the two hospitals during the observation period, including those who received prenatal care within the hospital system and/or those who gave birth at either hospital (Supplemental Table 1). Patients with gaps in healthcare utilisation were identified, using the Adequacy of Prenatal Care Utilisation (Kotelchuck)† index to categorise “less than adequate” prenatal care. Gaps in care, such as late prenatal care (initiated after six months), no postpartum follow-up and no paediatric follow-up within the hospital system, were also examined. Bivariate analyses were conducted among those who gave birth at either hospital, comparing characteristics of women with “adequate” or “adequate plus” prenatal care with women who had gaps in prenatal care (defined as “no care”, “inadequate” or “intermediate” care) within the hospital system. Chi-squared tests were used to compare characteristics, including age, race/ethnicity, neighbourhood, primary language, medical conditions, social services identified, insurance status at delivery, and self-reported alcohol and tobacco use during pregnancy. Multiple log-binomial regression estimated risk ratios associating patient characteristics with gaps in prenatal care, using the largest subgroup in each variable as the referent group. All quantitative analyses were conducted using SAS version 9.4, with *α* = 0.05 for all statistical tests.

### Qualitative data analysis

All interviews and focus groups were recorded, transcribed and translated into English if conducted in another language. Data were coded using Dedoose analysis software. Each transcript was individually reviewed by two analysts, who independently devised codes. These codes were then shared, and through iterative discussions, they were refined to create a final list of codes. The two researchers applied these finalised codes to all transcripts. Subsequently, they thematically analysed the coded data to identify cross-cutting themes that collectively characterised the interviews.

### Integration of quantitative and qualitative data

To provide a comprehensive understanding of maternal healthcare experiences and outcomes, we integrated the quantitative and qualitative findings using a convergent parallel mixed-methods approach, guided throughout by the Person-centred Care (PCC) framework. The PCC framework informed the selection of variables and outcomes in the quantitative component, including measures of prenatal care utilisation, gaps in care and maternal-infant health outcomes, ensuring alignment with principles of person-centered care. It also structured the qualitative component, guiding the development of interview and focus group questions to explore barriers, facilitators and perceptions of care across the maternal healthcare continuum. Integration occurred both analytically and interpretively. Quantitative analyses identified patterns of disparities and gaps in care, which were contextualised through qualitative findings from postpartum women, clinical staff and community-based organisation representatives. Convergent and divergent findings are presented in the Results section to demonstrate where quantitative trends aligned with qualitative themes and where they differed.

## Results

### Quantitative results

Characteristics of the EHR study population: Supplemental Table 1 summarises key characteristics of all women who were at least 18 years old and identified as pregnant (*n* = 5330) at the two hospitals between 1 January and 31 December 2019. The majority were women of colour, with White non-Hispanic women (*n* = 121) only comprising 2.3% of the total. Close to 40% of all women identified as Hispanic (*n* = 2216), followed by 20% who identified as Asian (*n* = 1059), and 17% who identified as Black or African American, non-Hispanic (*n* = 883). The two primary languages spoken by patients were English (*n* = 2876, 54%) and Spanish (*n* = 1824, 34.2%). Patients’ neighbourhoods were mainly from within the hospitals’ catchment areas, with a smaller proportion of patients from other NYC boroughs and an even smaller proportion from outside NYC and other states.

Of the 5330 women identified as pregnant at the two hospitals, 4074 (*n* = 76.4%) had at least one prenatal visit within the hospital system and 3181 (60%) gave birth at either hospital. Most women with prenatal visits within the hospital system were screened for depression using the PHQ-9 (except *n* = 107, 2.7%) and had their BMI recorded (except *n* = 33, 0.8%) during prenatal care. Nearly a third of women who had prenatal visits (*n* = 1212, 29.8%) were categorised as obese, i.e. BMI ≥ 30, and 18% (*n* = 745) had a history of diabetes. Self-reported alcohol use during pregnancy was 6% (*n* = 240), tobacco use was 1.6% (*n* = 63), and substance use was 3.1% (*n* = 128) among the 4074 women who received prenatal care within the hospital system. Almost all women who gave birth at either hospital had public insurance (*n* = 2875, 90%) at delivery, and the majority were multiparous (*n* = 1978, 62%). The preterm birth rate was 10% (*n* = 319) of all live births (*n* = 3202) at both hospitals.

#### Gaps in healthcare utilisation

Among the 5330 women registered as pregnant at the two hospitals, 920 (17.3%) were lost to follow-up, which we defined as having had no prenatal care visit or delivery recorded within the hospital system. Among those who gave birth at either hospital (*n* = 3181), 1502 (47.2%) did not initiate care in the first trimester, while 610 (19.2%) started prenatal care after six months of gestation, and 265 (8.3%) had no prenatal care recorded within the hospital system. Based on the adequacy of prenatal care utilisation (Kotelchuck) index, nearly half of those who gave birth had “adequate” (*n* = 1241, 39%) or “adequate plus” (*n* = 292, 9.2%) prenatal care. Of note, most women (*n* = 2898, 91.1%) did not have any primary care visits within the hospital system in the 12 months preceding their pregnancy. Further, 40% of women (*n* = 1267) had no postpartum follow-up visit within the hospital system after delivery. In contrast, only 5% (*n* = 159) of the 3162 live births at both hospitals had no paediatric follow-up visits within the hospital system. For the infants who did have at least one paediatric visit at NYC Health + Hospitals (*n* = 3003), the median number of visits was six [Q1, Q3: 3, 8]. Table 1 describes the gaps in healthcare utilisation within the hospital system.

**Table 1.** Gaps in health care utilisation in two public hospitals in New York City

Health care utilisation	Patients *n* (%)
Registered pregnancy at either hospital and lost to follow-up (*n* = 5330), *n* (%)	920 (17.3%)
Among women who gave birth at either hospital (*n* = 3181), *n* (%):	
No primary care visit in the NYC H + H system within 12 months before pregnancy	**2898** **(91.1%)**
Late prenatal care (after sixth month) in the NYC H + H system	610 (19.2%)
No prenatal care was initiated at NYC H + H within the first three months of pregnancy	1502 (47.2%)
Adequacy of Prenatal Care Utilisation (Kotelchuck) index	
*No prenatal care*	265 (8.3%)
*Inadequate (received less than 50% of expected visits)*	999 (31.4%)
*Intermediate (50%−79%)*	384 (12.1%)
*Adequate (80%−109%)*	1241 (39.0%)
*Adequate Plus (110% or more)*	292 (9.2%)
No postpartum follow-up within the NYC H + H system	1267 (39.8%)
No paediatric follow-up at NYC H + H hospitals among livebirths at either hospital (*n* = 3162), *n* (%)	159 (5.0%)

#### Characteristics of patients with gaps in prenatal healthcare utilisation

Chi-squared tests compared characteristics of the 1648 women (51.8%) who received no or inadequate prenatal care with the 1533 women (48.1%) who received adequate or adequate plus care within the hospital system (Table 2). Age, race/ethnicity, primary language, neighbourhood, social work visits during pregnancy, insurance type at delivery, delivery hospital, and self-reported tobacco use and alcohol use during pregnancy each appeared to be associated with gaps in healthcare utilisation in the hospital system. Women with adequate prenatal care within the hospital system tended to be older (≥ 30 years) and from neighbourhoods that were closer to the hospitals.

**Table 2:** Prenatal care utilisation (Kotelchuck index) among women who gave birth at two public hospitals in New York City by patient characteristics (*n* = 3181)

Characteristics	Patients who received adequate care at NYC H + H (*n* = 1533)	Patients with gaps^a^ in health care utilisation at NYC H + H (*n* = 1648)	*p*-value
**Sociodemographic characteristics**			
**Age, *n* (%)**			<.0001
**18–19 years**	36 (36.4%)	63 (63.6%)	
**20–24 years**	252 (40.1%)	376 (59.9%)	
**25–29 years**	427 (47.3%)	476 (52.7%)	
**30–34 years**	436 (51.4%)	412 (48.6%)	
**35–39 years**	297 (53.5%)	258 (46.5%)	
**40 years or older**	85 (57.4%)	63 (42.6%)	
**Race/Ethnicity, *n* (%)**			<.0001
**Asian**	416 (57.7%)	305 (42.3%)	
**Black or African American, non-Hispanic**	150 (32.2%)	316 (67.8%)	
**Hispanic**	719 (52.8%)	643 (47.2%)	
**Other**	198 (41.9%)	274 (58.1%)	
**Unknown**	19 (19.4%)	79 (80.6%)	
**White, non-Hispanic**	31 (50%)	31 (50%)	
**Primary language, *n* (%)**			
**Arabic**	7 (13.2%)	46 (86.8%)	<.0001
**Bengali**	124 (60.8%)	80 (39.2%)	
**English**	703 (44.3%)	883 (55.7%)	
**French**	6 (20%)	24 (80%)	
**Haitian Creole**	12 (35.3%)	22 (64.7%)	
**Hindi/Urdu**	31 (52.5%)	28 (47.5%)	
**Other**	41 (51.9%)	38 (48.1%)	
**Spanish**	609 (53.6%)	527 (46.4%)	
**Social work visits during pregnancy, *n* (%)**			0.0007
**None recorded**	1382 (47.3%)	1540 (52.7%)	
**At least one**	151 (58.3%)	108 (41.7%)	
**Insurance type at delivery, *n* (%)**			<.0001
**Self-pay**	4 (9.5%)	38 (90.5%)	
**Private**	158 (58.5%)	112 (41.5%)	
**Public**	1371 (47.8%)	1498 (52.2%)	
**Tobacco use, *n* (%)**			<.0001
**Never used**	1453 (49.4%)	1490 (50.6%)	
**Smoker (during pregnancy)**	10 (30.3%)	23 (69.7%)	
**Former smoker**	63 (41.7%)	88 (58.3%)	
**Missing**	7 (13%)	47 (87%)	
**Alcohol use during pregnancy, *n* (%)**			<.0001
**No**	1397 (49.4%)	1433 (50.6%)	
**Yes**	98 (56.3%)	76 (43.7%)	
**Missing**	38 (21.5%)	139 (78.5%)	
**Parity, *n* (%)**			0.970
**Nulliparous**	538 (48.3%)	576 (51.7%)	
**Multiparous**	949 (48.1%)	1024 (51.9%)	
**Missing**	46 (48.9%)	48 (51.1%)	
**Outcomes**	**Mothers received adequate care at NYC H** **+** **H (*n*** **=** **1527)**	**Mothers with gaps in health care utilization at NYC H** **+** **H (*n*** **=** **1630)**	***p*-value**
**Preterm births among mothers who had livebirths, *n* (%)**			
**Preterm**	141 (48.6%)	149 (51.4%)	0.930
**Term**	1386 (48.3%)	1481 (51.7%)	
**Low-birth weight among livebirths, n (%)**			
**No**	1388 (48.4%)	1481 (51.6%)	0.840
**Yes**	139 (47.8%)	152 (52.2%)	
^a^We defined a health care gap as having a Kotelchuck index of 0, 1 or 2, i.e. no prenatal care, inadequate (less than 50% of expected visits), or intermediate care (50–79% of visits).			

Black or African American women, Arabic-speaking women and those who reported using tobacco during pregnancy appeared more likely to have gaps in healthcare utilisation within the hospital system. However, given the small sample sizes in subgroups, these results need to be interpreted with caution.

The log-binomial regression includes the following variables: age, race/ethnicity, language, social work visit during pregnancy, insurance type at delivery, tobacco and alcohol use during pregnancy, and parity (Table 3). Results indicate that younger mothers (18–19 years) had 1.89 times the risk of having gaps in prenatal healthcare utilisation (95% CI: 1.17, 3.06) compared to the referent group (25–29 years), keeping all other variables constant. Of note, Black or African American, non-Hispanic mothers had 1.5 times the risk of having gaps in their prenatal care compared to their Hispanic counterparts (95% CI: 1.1, 2.1), while those with “Unknown” race/ethnicity had 3.2 times the risk (95% CI: 1.82, 5.5). There were higher gaps in prenatal care utilisation among Arabic-speaking women compared to those whose primary language was English (RR: 5.826, 95% CI: 2.5, 13.59), as well as among French-speaking women (RR: 2.572, 95% CI: 1.01, 6.56), although the sample sizes in these subgroups were small. Women with private insurance had a lower risk of inadequate prenatal care utilisation compared to those with public insurance (RR: 0.62, 95% CI: 0.47, 0.82), while those categorised as “self-pay” in the EHR had a higher risk of gaps in prenatal healthcare utilization (RR: 5.7, 95% CI: 1.94, 16.52). Those with missing tobacco (RR: 3.902, CI: 1.55, 9.8) or alcohol use (RR: 3.1, CI: 2.05, 4.69) had a higher risk of gaps in prenatal care utilization, suggesting that lack of disclosure of substance use status may be a risk factor.

**Table 3.** Estimated risk ratios of having a gap in prenatal healthcare utilisation within the NYC H + H system using log-binomial regression.

Characteristic		Adjusted Risk Ratio (Point estimate)	95% Confidence interval
Age	18–19 years vs 25–29 years	1.893	(1.17, 3.06)
	20–24 years vs 25–29 years	1.34	(1.07, 1.68)
	30–34 years vs 25–29 years	0.86	(0.7, 1.06)
	35–39 years vs 25–29 years	0.796	(0.63, 1.01)
	≥ 40 years vs 25–29 years	0.656	(0.45, 0.96)
Race/ethnicity	Asian vs Hispanic	0.97	(0.72, 1.31)
	Black or African American, non-Hispanic vs Hispanic	1.522	(1.1, 2.1)
	Other vs Hispanic	1.094	(0.82, 1.47)
	Unknown vs Hispanic	3.158	(1.82, 5.5)
	White, non-Hispanic vs Hispanic	0.557	(0.3, 1.03)
Language	Arabic vs English	5.826	(2.5, 13.59)
	Bengali vs English	0.806	(0.57, 1.14)
	French vs English	2.572	(1.01, 6.56)
	Haitian vs English	0.867	(0.39, 1.96)
	Hindi/Urdu vs English	0.941	(0.54, 1.64)
	Other vs English	1.08	(0.65, 1.79)
	Spanish vs English	0.871	(0.67, 1.13)
Social work visit during pregnancy	0 vs 1	1.283	(0.97, 1.7)
Insurance type at delivery	Private vs Public	0.615	(0.47, 0.81)
	Self-pay vs Public	5.661	(1.94, 16.52)
Tobacco use during pregnancy	1 – Smoker vs 0 – Never	2.441	(1.04, 5.73)
	2 – Former vs 0 – Never	1.247	(0.87, 1.79)
	3 – Missing vs 0 – Never	3.902	(1.55, 9.8)
Alcohol use during pregnancy	Missing vs No	3.1	(2.05, 4.69)
	Yes vs No	0.694	(0.5, 0.98)
Parity	0 vs 1	0.835	(0.7, 0.99)

### Qualitative results

Thematic analysis unveiled various barriers to maternal healthcare delivery and identified potential solutions across the three PCC domains: Social Determinants of Health, Health-Seeking Behaviours and Facility Quality. The qualitative sample consisted of 17 key informant interviews – nine with clinical staff (including an OB/GYN resident, physician assistant, attending OB/GYN, nurse administrator, midwife, primary care physician, clinical psychologist, licenced clinical social worker and director of nursing) and eight with representatives from our partner CBOs including programme managers, translators, directors, community health workers, case managers and maternal health advocates. All but one key informant were women. Additionally, five focus group discussions were conducted with 20 postpartum women. While we did not collect detailed demographic data in order to preserve participant anonymity, participants represented a diverse range of racial, ethnic and linguistic backgrounds, including individuals who spoke English (*n* = 3), Spanish (*n* = 5), Bengali (*n* = 4), Mandarin (*n* = 2) and Nepali (*n* = 6).

**Figure 1.**
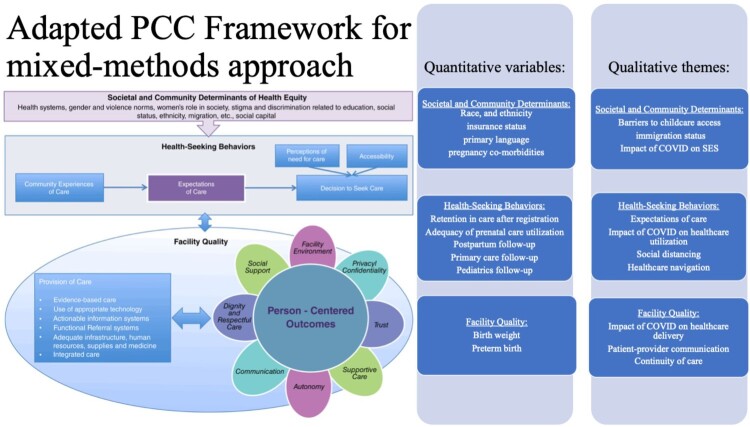
Adapted Person-Centered Care Framework for reproductive health equity

In the subsequent sections, we emphasise the most significant themes. For a comprehensive overview of each theme, its definition and additional supporting quotes, please consult Supplemental Table 2 in the appendix. Refer to Figure 1 for a comprehensive list of the quantitative variables and qualitative themes extracted based on the PCC framework.

### Domain 1: social determinants of health

Within the Social Determinants of Health domain, the most salient themes were lack of childcare impacting healthcare utilisation, stress related to immigration status and limited financial resources. Postpartum women, clinicians and members of community-based organisations consistently underscored the heightened challenges associated with inadequate childcare access, impacting the utilisation of quality healthcare services. A female midwife in a KII articulated, “*I think, from what I can tell, the main issue, besides the financial, is the childcare. They don't come because there's nobody else who can watch their kids*.” The recurrent theme of being unable to access healthcare services due to childcare limitations echoed across all interviews. Another female midwife elaborated*, “Maybe their childcare access as well. Because sometimes that'll hold people back from seeking care. Yeah, if they don't feel they can bring their kids, they don't have anywhere to leave their children.*” This consistent barrier to healthcare access highlights the critical need for supportive policies that address childcare availability, particularly for postpartum women. As the data show, when childcare is unavailable or unreliable, it directly prevents individuals from seeking necessary medical care, thus exacerbating health disparities.

The second thematic strand, revolving around the apprehension stemming from immigration status and related experiences, permeated various interviews. A representative insight was shared by a CBO director during a KII, who articulated,

“*We do have a large undocumented population that we work with. And a lot of times, there is a lot of fear among using public health benefits, public benefits. So, we do teach them, we do explain to them, like what are the benefits. I think a lot of them get worried that it is going to affect their immigration status if they ever apply for a permanent resident card, or if they are an asylee, how it's going to impact, you know, their future.*”

The fear linked to immigration status includes concerns about both immediate implications and the long-term repercussions on individuals’ immigration journeys and overall well-being. This fear is compounded by socioeconomic vulnerabilities associated with undocumented status. As one participant in the Spanish focus group explained, *“Due to our undocumented status, we earn less than minimum wage. Most pregnant women need to work. Because we do hard work through pregnancy, there is a risk of an accident or problem with the baby’s development.”* This account underscores the intersection of immigration status, economic precarity, and occupational health risks, particularly during pregnancy. It illustrates how immigration-related stressors extend beyond healthcare access to shape everyday working conditions, with direct consequences for maternal and fetal health.

The final theme, limited financial resources, was observed within the community prior to the pandemic and was further compounded by the financial crisis that followed. A poignant insight was shared by a social worker, illuminating the financial upheavals faced by many patients. A female social worker articulated during a KII,

“*You know, there's a big financial crisis. A lot of patients are undocumented; they don't qualify for any kind of stimulus package, or they've lost their jobs. Most of them are coming from areas like restaurants, laundromats, nail salons; their financial connections were cut. So, finances are a big crisis. I have a lot of women who are about to deliver and have nothing for their babies. The resources that we used in the past, such as Bridge to Life, and other resources, were closed.*”

This sentiment was echoed by a participant in the Nepali focus group, who shared,

“*This has affected our financial status because we haven’t been able to work since March; we had to stay home. As a result, we’ve had financial difficulties, but the government relief package helped us through the crisis. Now that everyone is back to work, it has made us realize the importance of financial security. We learned the necessity of keeping an emergency fund, with 3–6 months’ worth of savings. If we hadn’t received the government relief package, our savings wouldn’t have been enough, and it would have been difficult, especially after delivery.*”

Despite these financial burdens, one participant in the Nepali focus group shared her concern, stating,

“*Although I am ready to work, I fear getting exposed to the virus through sneezing or other means and passing it on to my 5-month-old baby. That’s why I only go out twice a month. Physically, I feel ready to work, but I am hesitant due to the risks.*”

This sentiment reflects the deep tension between financial survival and the need to protect both personal and public health. The pandemic not only amplified the financial disparities within vulnerable communities but also forced individuals to make difficult decisions that impacted both their economic stability and their families’ well-being.

### Domain 2: health-seeking behaviours

The themes related to health-seeking behaviours included: mismatches between patients and providers around expectations of care, challenges in navigating the US healthcare system as an immigrant, and the impacts of COVID-19 on healthcare utilisation. During focus group discussions, participants articulated their expectations from clinical providers and how these expectations influenced their decisions to seek care. One participant in a Mandarin FGD expressed, “*It’s generally alright, but the waiting time for check-ups was ridiculously long, more than an hour”* reflecting dissatisfaction with logistical barriers in the care experience. Conversely, a female physician assistant offered a contrasting perspective, stating,

“*Sometimes I think they have unrealistic expectations … I think their health literacy affects their perception. You know, they'll watch TLC birthing stories, which are all beautiful … but it also can be very complicated, bad things can happen. Right? Not that I want to focus on the bad things, but, um, I think sometimes they, they're not reading up during their pregnancy and we're not educating them.*”

This dichotomy in perspectives reveals the complex interplay between patient expectations, wait times and health literacy, highlighting the critical need for clear communication and patient education to align expectations with clinical realities. While the provider’s comment reflects a single perspective, it surfaces a broader tension in maternal care: how cultural context, access to information, and system navigation shape differing understandings of what quality care should look like. Rather than reducing these experiences to individual misunderstandings, this theme underscores the importance of culturally responsive care and mutual dialogue. These disconnects are not merely interpersonal – they point to deeper structural and quality of care challenges embedded in a system that often fails to equitably meet the needs of immigrant and marginalised communities.

The second theme affecting health-seeking behaviour was the challenge in navigating the US healthcare system as an immigrant. One patient, a foreign-born immigrant in a Nepali-speaking FGD, recounted, *“Since we are new and don’t know many processes in the hospital, I would recommend having a doctor who can speak my language. That way, we could share more about our experience.”* Additionally, a female CBO director during a KII shared:

“*It was her first OB-GYN visit in the United States, and she had no idea what she was in for. She said the doctor never explained what the procedure was or anything, and it was extremely painful … Nobody told her what that visit entails, you know, things like that. So, I think they need more education before they go.*”

The context of this quote suggests a lack of informed consent and pre-visit education, both of which are essential components of quality care. The patient’s pain may not have stemmed solely from the physical procedure, but also from the fear, confusion, and lack of preparation that accompanied it, highlighting a serious breakdown in communication and trust. Another female nurse director further emphasised these challenges, stating,

“*I have a mother who has dementia and who's 93, and navigating some of her services is not easy for me, and I'm in the field. If you have English as a second language, even if it's your first language, sometimes it's not easy with the necessary steps to access some of the programs you need and paperwork you have to complete.*”

These accounts illuminate the complexities inherent in navigating the US healthcare system, emphasising the critical need for improved patient education, clinical cultural competency training and streamlined processes to facilitate access, particularly for individuals with language barriers or varying degrees of health literacy.

Finally, the COVID-19 pandemic significantly affected care-seeking behaviours due to fears of contracting the virus at healthcare facilities and concerns regarding maintaining social-distancing measures. One female midwife stated in a KII:

“*We were obviously hit very hard at [redacted], and we were trying to talk to people and convince them that it was still safe to come. So a lot of it, in the beginning, was people dismissing their appointments. All they're seeing on the news is [redacted] with the freezer trucks of bodies and the emergency room full of people … We found ourselves calling everyone who missed their visit, trying to reassure them that they can still come to the clinic.*”

A participant in the Mandarin focus group stated that,

“*The problem is, I am rather nervous. I am scared because my body is quite weak after giving birth this time, and I have two kids to care for. The positive side is that there are no visits. Friends do not come to visit me and the kids, so it’s relatively safe. What makes me even more nervous is that I am so worried that my kids or I would get sick, but I won’t dare to go to the hospital or clinic.*”

Another participant in an English FGD shared a deeply personal account of fear and grief:

“*A month after my daughter was born, I lost my uncle to COVID, my mom and my dad got sick. So to me, the fear that I might lose my mom or my dad – that's been my support system, 100%. They've always had my back in everything. To me, it was overwhelming. And now, to be honest with you, with the whole situation going on … I don't come out, I don't go out at all. I don't try to expose myself. I buy online, everything I need. If I have to go to a doctor's appointment, that's as far as I go. You know, because I think about my little ones. After losing my uncle, it's just like a fear that I have. Not so much a depression, but a fear.*”

This contrast highlights a dissonance in the perception of risk between providers and patients. While the clinical staff member was focused on reassuring patients and maintaining continuity of care, their efforts to encourage in-person visits may have inadvertently underestimated the very real and immediate fears patients faced – especially in the early days of the pandemic, before vaccines were available. For postpartum individuals managing both physical recovery and caregiving responsibilities, the fear of COVID-19 exposure posed a legitimate threat to their health and family stability. This underscores the importance of risk communication that is empathetic and responsive to patients’ lived realities, particularly in times of public health crisis. As a female OBGYN said, “*Just like systemic racism is so ingrained … you don’t see it. It’s like the refrigerator is on and you don’t hear it … it needs to be looked at. I think people don't hear it, because it's so common that we just don't see it.”* This statement underscores how deeply embedded structures of inequality – such as racism – often go unnoticed by those in the system, despite their pervasive influence on patient-provider interactions. It reflects how healthcare environments may unintentionally reinforce systemic inequities, making it even more critical for health systems to actively address these invisible but real barriers. As such, health-seeking behaviours are not only shaped by logistical and informational challenges but also by the social and institutional forces that may make some individuals feel invisible or marginalised within the healthcare system.

### Domain 3: facility quality

Within the third domain, patient-provider communication, disruptions in the continuity of care and the impact of COVID-19 on healthcare delivery emerged as predominant themes. Participants, both patients and clinical professionals, conveyed the profound impact that patient-provider communication had on the delivery and receipt of care, particularly within a predominantly immigrant community marked by diverse cultural differences. In a poignant reflection from a focus group discussion, one participant shared in the English FGD,

“*But the nurses were there for me, you know, more than my own doctors … I feel like doctors don't pay much attention to their patients, because they're so used to people having these experiences. They think everyone is in the same boat. And the nurses, they're just more so like, ‘Yes, like, Oh my gosh, we want to help.’ You know, they do anything they can to help more so than your own doctors.*”

This account highlights the disparity in attention and empathy between doctors and nurses, shedding light on the unique role nurses play in providing personalised and compassionate care.

Continuity of care was highlighted as both a challenge and a facilitator. During an FDG, one Mandarin-speaking participant noted, “*Each check-up, I was examined by a different doctor … not sure if it’s related to the hospital or its maternity unit, but I felt that experience wasn’t very good. It’s because my first kid was delivered by my own obstetrician.*” In contrast, another participant in a Bengali FGD praised her provider:

“*She knew all my history, so she supported me a lot. By looking at my face, she even memorized my name. Whenever I entered, she greeted me by saying, ‘Oh, you’ve come. Okay, come; please come to my room.’ It was such a wonderful experience for me. I didn’t need to wait there. Whenever she saw me, she called me instantly.*”

A clinical psychologist during a KII noted a lack of continuity due to patients’ presenting late or transferring care:

“*We sort of lose track of them, or they end up moving somewhere else … So that's hard. Especially when we get patients who come in here during their third trimester or transfer care out during their third trimester. Recently, we've had patients who, like, their delivery was their first time in this hospital.*”

These contrasting narratives underscore the critical role continuity of care plays in shaping patient trust, satisfaction and overall experience, particularly during the perinatal period.

The COVID-19 pandemic also affected the perceived quality of care in healthcare facilities, particularly due to the widespread shift to video and phone telehealth. Many patients felt that virtual visits altered the level of care they received, especially during the postpartum period. During the Spanish-language FGD, participants described collective challenges related to the impact of COVID-19 on postpartum care quality. They conveyed a shared sense of isolation and emotional distress during the immediate postpartum period, citing hospital restrictions that limited in-person support and, in some cases, access to their newborns. These constraints exacerbated feelings of stress and loneliness during childbirth and recovery. When asked if the quality of services received through video telehealth was affected, a mother in the Nepali focus group said, *“It’s not the same as physical consultation – it feels like something is missing.”* Similarly, a participant in the Spanish focus group shared, “*If you make a telehealth visit, it’s very basic and quick.”* Clinicians also shared their perspectives. A female clinical psychologist during a KII provided valuable insights, sharing,

“*I think when it first started, I was probably doing close to 90% telehealth. The phone can be really difficult if patients aren't focused and it's really limiting our ability to interpret their nonverbal communication. And with video, some people just don't feel comfortable doing it or feel like they don't want you to see their view. They're ashamed of their home environment. But for some people, video works great; they feel comfortable and would rather do video than come in person.*”

A female OBGYN echoed this sentiment, saying,

“*I'm not a huge fan. Calling a patient I've never met and asking about their sexual history over the phone can feel inappropriate. It's hard when you have to use an interpreter. We also weren't using video calls; it was just a straight phone call. So you're not seeing the face and really seeing the body language or what's going on. So, it was a little awkward, I'd say video health would be a better choice just for the rapport, you know, the connection.”*

In contrast, a male primary care provider reflected on the benefits of telemedicine, stating,

*“While we did a lot of telemedicine at first, we’ve found that for many, in-person visits are still necessary. However, for those who can be served remotely, it’s better not to make them travel, especially considering transportation and parking issues.”*

These insights highlight the mixed experiences with video and phone telehealth: while it offered increased accessibility for some, for others it lacked the depth and connection of in-person visits. In the context of postpartum care, where trust, comfort, and physical assessments are often vital, the shift to virtual services introduced significant challenges in maintaining care quality and emotional support.

## Mixed-methods integration

Exploring person-centred care across the domains of Social Determinants of Health (Domain 1), Health-Seeking Behaviours (Domain 2) and Facility Quality (Domain 3) revealed how quantitative and qualitative findings converge and diverge, offering a nuanced understanding of challenges and opportunities in maternal/child healthcare delivery in NYC. Our mixed-methods design allowed us to triangulate data across three perspectives – postpartum women, clinical providers and CBO staff – highlighting both alignment and divergence in priorities and experiences.

### Domain 1: Social determinants of health

Qualitative results in the first domain, Social Determinants of Health, highlight significant barriers to childcare access, exposing the profound impact that inadequate childcare can have on healthcare utilisation. The theme resonated across various perspectives: postpartum women, clinicians and community-based organisation members. This finding is supported by our quantitative data, which indicates that nearly 40% of individuals identified through EHR data did not attend a postpartum follow-up visit within the NYC H+H system, demonstrating convergence between qualitative findings and quantitative indicators of missed postpartum follow-up. Furthermore, stress related to immigration status emerged as a prominent theme in this domain, underscoring the need for a safe and supportive healthcare environment, particularly for vulnerable communities of colour. Additionally, this domain highlighted how the COVID-19 pandemic has significantly affected the socioeconomic status of patients, leading to financial crises, loss of jobs and reduced access to resources, particularly for undocumented individuals. In our study, related quantitative analysis showed that individuals with private insurance (a proxy for higher socioeconomic status) had a lower risk of having a gap in prenatal healthcare utilisation (RR: 0.62, 95% CI: 0.47, 0.81) compared to those with public insurance, suggesting that those ineligible or unable to access benefits face the steepest care barriers to consistent and adequate maternal healthcare.

### Domain 2: Health-seeking behaviours

Mismatches in expectations of care, one of the focal themes within the second domain of Health-seeking behaviours, reveal the complex dynamics of the patient-–provider relationship. The dichotomy between patient expectations and the realities of healthcare experiences, as evidenced by clinicians and patients themselves, calls for a paradigm shift in communication and education. This tension emerges at the intersection of qualitative accounts of confusion and quantitative evidence showing delayed initiation or transfer of prenatal care – both pointing to significant discontinuity in care. Quantitatively, our data show that over half (51.8%) of individuals within the H+H system lacked adequate prenatal care, suggesting that delayed presentation or care transfers may contribute to these gaps. Our qualitative data further reveal the substantial challenges immigrants face when navigating the US healthcare system, often compounded by fear, confusion, and inadequate preparation.

### Domain 3: Facility quality

In the final domain, Facility Quality, video and phone telehealth emerged as both a critical adaptation and a notable challenge. While it ensured continuity of care during the COVID-19 pandemic, it also introduced barriers for many patients, particularly immigrants with limited digital literacy or English proficiency. Participants described discomfort with virtual platforms, confusion navigating new technologies, and missed emotional cues in the absence of in-person interaction and interpretation.

Building on this, while our quantitative analysis revealed group-specific disparities – such as a higher risk of inadequate prenatal care among Black or African American women – the qualitative findings uncovered more universal themes that cut across demographic lines. We did not observe major differences in reported experiences by ethnicity or language; however, we identified areas of both convergence and divergence, particularly in perceptions of provider communication, system navigation, and emotional support.

## Discussion

Findings from this mixed-methods study reveal how structural, social and institutional factors intersect to shape maternal and postpartum healthcare utilisation and person-centred care experiences within NYC public hospitals, particularly during the COVID-19 pandemic. Exploring the Person-Centred Care framework across three domains – Social determinants of health, Health-seeking behaviours and Facility quality – revealed both shared priorities and areas of divergence. Notably, this work is unique in its approach of examining issues and solutions through three different perspectives – clinical providers, CBO staff and postpartum women – highlighting where priorities align, where they diverge, and how those insights can inform more equitable, person-centred care models.

Our findings are congruent with existing literature: Black or African American mothers appear to have more gaps in prenatal healthcare, and urban US studies have demonstrated that immigrant mothers face gaps in recommended peripartum and postnatal care.^10^ However, this study suggests that Arabic-speaking women comprise a potentially high-risk subgroup, which is less frequently disaggregated in existing studies on immigrant health. Given the small sample sizes for this subgroup in our study, more research is needed to elucidate potential disparities. Results also echo prior work linking immigrant status to limited public insurance eligibility, fear of immigration enforcement and confusion around benefits, all of which constrain postpartum care access. A pre-pandemic 2020 study examining the association between migrant women’s legal status and prenatal care utilisation in France found that all groups of migrant women were at greater risk of inadequate prenatal care utilisation (PCU) compared to French-born women (26.4%), with rates of 31.6% among legal migrants with European nationalities, 40.3% among other legal migrants and 52.0% among undocumented migrants.^21^

These findings reinforce calls to integrate structural determinants into person-centred frameworks. A scoping review of US-based studies examining pregnancy care among undocumented populations found that immigration status, alongside exclusionary policies and rhetoric, is associated with lower care utilisation and poorer pregnancy outcomes. Conversely, inclusive healthcare and immigration policies were linked to improved prenatal and postnatal care utilisation and better maternal outcomes.^22^ A study analysing data from six US states between 2020 and 2022 found that non-citizen women without permanent resident status had substantially lower rates of postpartum visits and health insurance coverage in the late postpartum period compared to US citizens.^23^ Similarly, a 2017 Kaiser Health Survey found that, in comparison to men, women were more prone to postponing or forgoing healthcare, with as many as 26% of women reporting delays in preventive services and skipping recommended tests or treatments with logistical barriers associated with women's roles as caregivers.^24^ Furthermore, 24% of women cited a lack of time for doctor visits, 23% faced challenges taking time off from work, and 14% experienced delays in their healthcare due to insufficient childcare.^24^ Although these obstacles impacted women across the board, low-income women were more prone to facing both childcare challenges and delays in accessing healthcare.^24^

Qualitative narratives uncovered deeper explanations for what might appear as disengagement, such as confusion about appointment logistics, dissatisfaction with prior care, or life instability related to housing and immigration status. These findings highlight how structural and social barriers intersect with relational factors, including communication quality, trust and cultural sensitivity, to shape healthcare experiences. Prior research shows strong links between clinician-patient communication and adherence; for instance, a review of 127 studies found positive associations in 125 cases, with adherence odds 1.62 times higher when physicians received communication training.^25^ However, existing frameworks inadequately address immigrant-specific factors, including the influence of acculturation on expectations and engagement.

Notably, the existing literature on expectations lacks a thorough exploration of the influence of acculturation, especially from a person-centred perspective.^26^ Each phase of an individual's journey can profoundly shape their healthcare-related expectations and outcomes, yet strategies to address these factors remain insufficient.^27, 28^ Insights from our qualitative interviews, using the PCC framework, highlight how mismatches in expectations – whether stemming from cultural or communication gaps – or concrete quality-of-care issues, such as long wait times or inconsistent providers, affect maternal care. These issues are not merely differences in perception but reflect structural and systemic challenges that demand targeted quality improvement interventions. We propose that mismatches in expectations be distinguished from actual quality of care issues, such as delays and cultural insensitivity, with each serving as a distinct opportunity for focused intervention. Beyond structural factors, qualitative findings highlight relational dynamics that influence patient experiences, including communication quality, trust, and cultural sensitivity. Prior literature shows that improved clinician-patient communication increases adherence and satisfaction, yet existing frameworks rarely incorporate acculturation and immigrant-specific challenges. By documenting how mismatches in expectations, discontinuity of care and facility-level barriers intersect, our study extends these findings and emphasises the need for multifaceted interventions, combining patient education, provider training and system-level redesign, to support equitable maternal care.

Facility-level findings highlight the dual nature of telehealth. While enabling continuity of care during COVID-19, virtual platforms introduced challenges for patients with limited digital literacy or privacy constraints. This calls for a nuanced approach to telehealth implementation, recognising and addressing the diverse preferences and barriers faced by patients. A qualitative study conducted in 2020–2021 with 44 immigrant women and 19 direct service providers in New York City found that many women struggled to access high-quality internet and unfamiliar virtual platforms, especially when privacy was limited.^29^ In-person visits were described as vital for surfacing concerns that might otherwise go unspoken. particularly around mental health or intimate partner violence.^29^ Some participants expressed fear or shame, noting that these issues were difficult or unsafe to disclose when visits took place in shared households or with aggressors nearby.^29^ These findings underscore that telehealth cannot be a one-size-fits-all solution and must be thoughtfully implemented with attention to individual needs and constraints.

Taken together, our findings suggest several avenues for intervention and policy innovation. Recognising the impact of social determinants means going beyond screening for needs to actively integrating supportive services into care delivery, such as offering on-site childcare or flexible appointment hours to accommodate working mothers, particularly those with limited support systems. Addressing immigration-related stressors may involve creating welcoming, linguistically and culturally responsive environments that include legal aid partnerships or dedicated care navigators for non-English speakers.

To align service delivery with patient expectations, communication training for providers should be emphasised, not only to improve care engagement but also to ensure providers are attuned to patients’ cultural beliefs and definitions of care quality. Mismatches in expectations – such as assumptions about what constitutes adequate prenatal or postpartum care – can be addressed by routinely eliciting patients’ goals and preferences during intake and follow-up visits, and by improving continuity of care, particularly for those who transfer into the system late in pregnancy.

A facility optimised for person-centred care, especially for immigrant and low-income populations, would offer integrated mother-infant visits during the postpartum period, as piloted at one study hospital. It would also leverage paediatric visits, which have high attendance, as touchpoints for maternal health assessments. Video telehealth platforms, when used, would be designed with attention to patients’ digital literacy and comfort, potentially offering hybrid models that allow patients to toggle between in-person and virtual care depending on their needs. Initiatives like the HOPE Doula Programme – a collaboration among NYC Health + Hospitals/Elmhurst and Queens, academic institutions and community-based organisations – demonstrate how free, culturally and linguistically relevant doula care can advance equity.^30^ By providing continuous, culturally attuned support across the perinatal period, the programme exemplifies person-centred, emotionally supportive maternity care that can be scaled nationwide to improve outcomes.^30^ These innovations are not only clinically effective; they are acts of reproductive justice. Facilities that embed such models bring core human rights principles to life, ensuring care is accessible, culturally and linguistically appropriate, and safe. By addressing both structural barriers and interpersonal dynamics, they move beyond service delivery to uphold the autonomy and dignity of marginalised birthing people. In this way, rights-based care is not aspirational but essential to effective maternal health.

### Strengths and limitations

Delivering person-centred care requires more than rhetoric; it demands redesigning systems to be flexible, inclusive, and responsive. Insights from this study point to actionable strategies that, if implemented, could make healthcare more relational, equitable and effective for the communities that need it most. Our triangulated mixed-methods design, anchored in the Person-Centered Care Framework for Reproductive Health Equity, allowed for meaningful integration of findings across domains, elevating both patient- and system-level perspectives. Framing our work within a reproductive health equity lens clarifies the structural and rights-based dimensions of these challenges and underscores the ethical imperative to centre the needs of historically marginalised communities in maternal health reform. The relatively large and diverse quantitative sample, paired with qualitative data from postpartum women, providers and community-based staff, helped surface critical dynamics often overlooked in maternal health research, particularly the intersecting challenges facing immigrant communities. These strengths deepen the relevance and potential impact of our findings, offering a foundation for future interventions that are both empirically grounded and equity-oriented.

This study is subject to several limitations. Despite our efforts to assemble a diverse group of postpartum women for focus group discussions, challenges in recruitment and enrolment hindered our ability to reach the recommended five participants in each group, even with the offered incentives. COVID-19–related social-distancing restrictions compelled us to resort to video interviews, a shift that, while necessary, might have hindered enrolment and changed the nature of the conversations. Additionally, while we successfully conducted interviews in various languages, translating the transcripts back to English by native speakers for Spanish, Nepali, Bengali, and Mandarin may have lost important cultural nuances, as meaning is more likely to be diminished rather than enhanced in translation.

Notably, no focus groups were conducted in Arabic, French, Haitian Creole, or Hindi/Urdu, and we did not partner with CBOs specifically serving these populations. This potentially represents a significant gap, especially considering that we draw conclusions about barriers to care that may uniquely impact this community. However, this population was not identified by our community partners as a high-need group within our immediate catchment area, which may have influenced recruitment priorities. Consequently, while our conclusions about barriers to care apply broadly across the represented groups, they cannot be fully generalised to these linguistic communities, whose experiences may differ in important cultural and systemic ways. We were also unable to provide childcare during the focus groups, which may have further deterred participation, particularly for low-income parents who often miss critical healthcare appointments due to childcare constraints. While participants were encouraged to take breaks as needed, the absence of structured childcare remains an important limitation to accessibility and inclusion.

Most key informant interviews were conducted with female-identifying providers and staff; only one male provider participated. This gender imbalance may limit the range of perspectives, particularly regarding gender dynamics in patient-provider interactions.

In addition, our use of EHR data for quantitative review also posed limitations. Some data were missing, especially for self-reported variables such as alcohol use during pregnancy. However, these were missing for less than 5% of the patient population and may have been underreported due to social desirability. Race and ethnicity categories also proved limiting; about 3% of patients were classified as “Unknown,” and the predefined categories may not have fully captured the complexity of patients’ identities. In our logistic regression, some of the confidence intervals were large, indicating greater uncertainty of the estimates, due to small sample sizes of some categories, such as “self-pay” and Arabic-speaking participants. Lastly, although we assessed healthcare utilisation within the NYC Health + Hospitals system, we could not account for care received outside the system or instances in which patients chose to discontinue care altogether.

Despite these acknowledged limitations, our findings offer a valuable evaluation of the healthcare system's impact on maternal healthcare delivery and usage before and during the COVID-19 pandemic. These insights offer a valuable foundation for future research and developing and refining system-level strategies that advance equity, particularly for immigrant and linguistically diverse communities.

## Conclusion

Maternal health inequities in the US have gained prominent national and local attention, emphasising the urgency of addressing disparities through systems-level change. To create impactful interventions in diverse communities, a nuanced understanding of community-specific barriers and social determinants is imperative. Grounded in the Person-Centered Care Framework for Reproductive Health Equity (PCC), our study approaches maternal health not only as a clinical concern but as one inherently linked to the fulfilment of reproductive rights and equity. The framework informed both our design and interpretation, enabling us to surface structural and relational dimensions of care that often go unaddressed in healthcare delivery. Our results corroborate existing data indicating that maternal health statistics are far worse for ethnic minorities. However, we also shed light on how these disparities were exacerbated by the pandemic, further impeding health delivery across social determinants, health-seeking behaviours and facility quality domains. These findings offer direction for future research that tests the framework’s applicability for reproductive and sexual health through the life course while also informing local and global strategies to advance person-centred, rights-affirming care.

## Supplementary Material

Supplemental Table 1: Characteristics of pregnant patients at two public hospitals in New York City.

Supplemental Table 2: Qualitative Themes with Accompanying Definitions and Supporting Quotes.

## Footnotes

* Throughout this paper, we use the terms women and birthing people to reflect both the language used in our data sources and our commitment to inclusive terminology. While the data analysed were collected using standard health records that categorise individuals as women, we recognise that not all individuals who give birth identify as women.

† The Kotelchuck Index, also known as the Adequacy of Prenatal Care Utilisation (APNCU) Index, evaluates prenatal care based on the timing of initiation and the number of visits received, adjusted for gestational age. It classifies care as inadequate, intermediate, adequate, or adequate plus

## Provenance statement

This article was not commissioned and went through external peer review.
